# Organ-Specific Phytochemical Profiles, Wound-Healing and Hemostatic Activities of *Symphytum officinale* Aerial Parts and Roots with Differential Pyrrolizidine Alkaloid Content

**DOI:** 10.3390/molecules31122159

**Published:** 2026-06-18

**Authors:** Getter Dolgošev, Yurii M. Kolesnyk, Olha Hancheva, Oleksandr Panasenko, Andrii Kaplaushenko, Roman Shcherbyna, Valdas Jakštas, Vaidotas Žvikas, Ivo Laidmäe, Jyrki Heinämäki, Oleh Koshovyi, Ain Raal

**Affiliations:** 1 Institute of Pharmacy, Faculty of Medicine, University of Tartu, 50411 Tartu, Estonia; ivo.laidmae@ut.ee (I.L.); jyrki.heinamaki@ut.ee (J.H.); ain.raal@ut.ee (A.R.); 2 Zaporizhzhia State Medical and Pharmaceutical University, 69035 Zaporizhzhia, Ukraine; kympat83@gmail.com (Y.M.K.); ganchevaolga1@gmail.com (O.H.); panasenko.o.i@zsmu.edu.ua (O.P.); kaplaushenko@ukr.net (A.K.); rscherbyna@gmail.com (R.S.); 3 Institute of Pharmaceutical Technologies, Lithuanian University of Health Sciences, LT-44307 Kaunas, Lithuania; valdas.jakstas@lsmu.lt (V.J.); vaidotas.zvikas@lsmu.lt (V.Ž.); 4 Department of Pharmacognosy, Lithuanian University of Health Sciences, LT-44307 Kaunas, Lithuania

**Keywords:** common comfrey, phenolic compounds, amino acids, pyrrolizidine alkaloids, hemostatic activity, wound-healing activity

## Abstract

Wound healing is a complex biological process involving inflammation, hemostasis, and tissue regeneration, and its impairment may delay recovery and lead to clinical complications. Medicinal plants have long been used in traditional medicine to support wound repair and control bleeding. Among these, *Symphytum officinale* L. (comfrey) is widely used in European traditional medicine for the treatment of wounds, fractures, and soft tissue injuries. However, the pharmacological basis of these effects and the contribution of different plant organs remain insufficiently understood. The aim of this study was to characterize the phytochemical composition of extracts obtained from different organs of *S. officinale* and to evaluate their hemostatic and wound-healing activities in vivo, in order to identify the most active plant parts. Extracts from the roots, leaves, and flowers of *S. officinale* were prepared using solvents of different polarity. Phytochemical composition was analyzed by spectrophotometric assays and UPLC–MS/MS. Hemostatic and wound-healing activities were evaluated in vivo in rats, and cytological analysis of wound exudate was performed. Distinct organ-specific differences in phytochemical composition were observed, with leaf and flower extracts richer in phenolic compounds and amino acids, while root extracts contained higher levels of pyrrolizidine alkaloids. Leaf (S7) and flower (S13) extracts showed the strongest biological activity. S13 accelerated wound healing, achieving complete wound closure by day 10 compared with day 12 in the reference group, while S7 exhibited the most pronounced hemostatic effect, reducing bleeding cessation time to 44.3 s compared with 64.1 s for the reference preparation and 144.3 s for the control group. Cytological analysis indicated reduced inflammation and enhanced fibroblast activity. The findings support the traditional use of *S. officinale* in wound treatment and highlight the importance of organ-specific phytochemical composition. Aerial parts showed strong wound-healing and hemostatic effects, suggesting their relevance alongside the traditionally used root and supporting the involvement of multiple constituents in the observed biological activity. The study provides new insights into the organ-specific phytochemical composition and biological activity of *S. officinale*, thereby supporting further research on the therapeutic potential of its aerial parts.

## 1. Introduction

The incidence of traumatic injuries continues to rise globally due to population ageing and the increasing number of crises and conflicts worldwide. As a result, the need for effective agents that support wound and bone healing, alongside growing interest in safe, natural therapeutic alternatives, has become increasingly prominent. *Symphytum officinale* (common comfrey, Boraginaceae) has a long history of use in traditional medicine for promoting wound and bone healing, as well as for alleviating muscle and joint pain [1,2].

The root is the most commonly utilised plant part, as it contains allantoin, a compound widely recognised for its wound-healing properties. Although allantoin levels in the leaves are considerably lower, leaf extracts have nonetheless demonstrated promising wound-healing activity, likely attributable to the presence of polysaccharides and other biopolymers [3,4]. Aerial parts have also been used medicinally. However, to the best of current scientific knowledge, the phytochemical profile and pharmacological effects of *S. officinale* flowers remain unexplored. Furthermore, no comprehensive organ-specific phytochemical comparisons of different *S. officinale* organs have been documented.

The biological activity of *S. officinale* has been investigated in bone regeneration. The plant has been shown to increase bone mineral density around titanium implants in rat tibiae [5]. A paste prepared from *S. officinale* tincture and calcium oxide was reported to support bone tissue regeneration and osteosynthesis [6]. Topical preparations derived from the root exhibited antimicrobial properties and accelerated wound-healing [7], while leaf extracts reduced inflammation and enhanced collagen deposition [8]. Clinical studies have also demonstrated its efficacy in reducing pain and oedema associated with ankle sprains [9]. Additionally, *S. officinale* herb extract shows α-glucosidase inhibitory activity, indicating potential relevance in the management of type 2 diabetes [10].

Chemically, *S. officinale* contains various amino acids, including histidine in the leaves; tyrosine and glutamic acid in the aerial herb; and methionine, arginine, and tyrosine in the roots [11]. Its polyphenolic profile includes rosmarinic acid, rabdosin, globoidnans A and B, *p*-hydroxybenzoic, caffeic, chlorogenic and *p*-coumaric acids, as well as ellagic acid [10,12,13]. Documented monosaccharides in the plant include arabinose, glucuronic and galacturonic acids, glucose, and galactose [14,15].

Toxicologically, *S. officinale* contains pyrrolizidine alkaloids (PAs), including intermedine, lycopsamine, their N-oxides, and C7-acetylated derivatives. In addition to these PAs, which are commonly found in leaves and roots, flowers contain myoscorpine and its C3-acetylated derivative, as well as symphytine isomer and its N-oxide [16,17,18]. Despite the presence of hepatotoxic PAs, topical preparations are considered relatively safe because dermal absorption of these alkaloids is limited [19].

Although *S. officinale* is a well-known medicinal plant, no comprehensive studies have investigated the phytochemical differences among its organs or their organ-specific pharmacological contributions to wound healing. In particular, comparative data on different plant organs and their roles in wound healing and hemostasis remain limited. Therefore, the aim of the present study was to investigate the organ-specific phytochemical composition of *S. officinale* and to evaluate, in vivo, the hemostatic and wound-healing activities of its extracts to better understand the pharmacological basis of its traditional use.

To the best of our knowledge, this is the first study to comparatively investigate the organ-specific phytochemical composition, pyrrolizidine alkaloid profiles, and wound-healing and hemostatic activities of different *S. officinale* plant parts, including flowers.

## 2. Results and Discussion

Although *S. officinale* is a widely used medicinal plant, several aspects of its phytochemistry and pharmacology remain insufficiently understood. The roots constitute the most commonly utilised plant part. However, comparatively little attention has been given to other organs, such as leaves and especially flowers. This study sought to address these gaps by examining the chemical composition of extracts from these less-studied plant parts and considering how their phytochemical profiles may influence their wound-healing potential.

The *S. officinale* extracts were dark green, brown or light brownish powders depending on the plant part and solution used in extraction, while polysaccharide fractions were grey. The yields of the dry extracts ranged from 11.18% to 33.54%.

### 2.1. Phytochemical Study

The phenolic compounds and amino acids in the *S. officinale* extracts were analysed by means of LC-MS (Table 1). In total, 12 phenolic compounds, 14 amino acids and 4 pyrrolizidine alkaloids were identified.

#### 2.1.1. Polyphenolic Compounds

In our study, we identified twelve phenolic compounds in the root, leaf and flower extracts of *S. officinale*. Rosmarinic acid (RA) and caffeic acid, as the main compounds, were detected across the extracts of all three plant parts. In the root extracts, the RA content was 4.23 mg/g (S3, 70% ethanol solution). The value is lower than previously reported (7.56 mg/g extract) [18], which may be due to differences in plant origin and extraction technique. However, in the flower and leaf extracts (extracted with 70% ethanol), the RA content was as high as 17.63 mg/g (S13) and 16.86 mg/g (S8), respectively. As stated in the findings of Kimel et al. [20,21], a higher content of RA was reported in the leaves of *S. officinale* in comparison to the roots. The range of RA content in the roots was between 0.59 and 1.84%, whilst in the leaves it was between 1.91 and 2.41%. It is important to note that there is a paucity of comprehensive data on the RA content in *S. officinale* flowers. It was determined that the RA amount in the tested flower extracts (70% ethanol solution) is only marginally lower than that in the leaves. This finding suggests that the utilisation of flowers, both in mixture with leaves and as a component of the above-ground herbal material, may be reasonable. It is particularly noteworthy that the high content of RA is of significance, given its strong antioxidant activity [18]. RA levels ranging from 1.23 to 5.05 mg/g have been reported in 65% aqueous ethanol extracts prepared from powdered material by sonication [22]. The study also showed how storage time affects the RA content in comfrey roots over 6 months. The results were similar to ours; in the root extracts, the RA value reached 4.23 mg/g (70% ethanol solution). As such, this phytochemical compound could be selected as a phytochemical marker for quality monitoring of related extracts.

Root extracts exhibited the lowest phenolic diversity, containing only caffeic acid and RA. In contrast, leaf extracts were the richest in phenolic compounds, comprising eleven distinct constituents. Other dominant polyphenols in leaves were isoquercitrin and caffeic acid. The phenolic composition of the flower extracts closely resembled that of the leaf extracts, with RA, isoquercitrin, and caffeic acid again representing the major components.

Aqueous extracts contained the highest levels of caffeic acid and *p*-coumaric acid compared to ethanolic extracts, although their concentrations of other phenolic compounds were generally lower. The extracts prepared with 40% aqueous ethanol showed intermediate overall phenolic levels. Notably, the highest concentrations of hyperoside and kaempferol-3-O-glucoside were found in the leaf extracts produced with 40% aqueous ethanol. The 70% aqueous ethanol extracts yielded the highest overall levels of diverse polyphenolic constituents. Importantly, the highest concentration of RA, the primary phenolic component, was found in the extracts prepared with 70% aqueous ethanol.

Caffeic acid has previously been detected in the root and leaf water extracts of *S. officinale* at 0.15 and 0.29 mg/g, respectively [23]. We found similar levels in the root water extracts (0.14 mg/g), whereas the concentration in the leaf extracts was substantially higher (1.21 mg/g). We also quantified caffeic acid for the first time in the flower extracts, where its concentration (0.82 mg/g in water extract) was comparable to that of the leaf extracts.

Leaf and flower extracts were also rich in isoquercitrin. To the best of our knowledge, this is the first study revealing isoquercitrin content across different organs of *S. officinale*. Moreover, we report for the first time the presence of hyperoside and several other phenolic constituents, such as phloridzin and 3,4-dihydroxybenzoic acid, specifically in leaf extracts.

In flower extracts, isorhamnetin-3-glucoside was also identified as part of the whole phytochemical profile of the material and preparations. Isoquercitrin and hyperoside, kaempherol-3-O-glucoside, isorhamnetin-3-glucoside, and ferulic acid were found as the characteristic compounds in *S. officinale* flower extracts.

#### 2.1.2. Amino Acids

A total of 14 amino acids were identified across the root, leaf and flower extracts of *S. officinale*. The extracts contained several essential amino acids, including histidine, isoleucine, lysine, methionine, and phenylalanine.

Root extracts exhibited the lowest diversity and the lowest amino acid concentrations. Histidine and lysine were the predominant constituents in the root extracts. In contrast, the leaf extracts were rich in essential amino acids, with isoleucine, leucine, histidine, and valine at the highest levels. The flower extracts showed the greatest overall amino acid abundance, with aspartic and glutamic acids as the most prominent components, while histidine, proline, and valine were also present at notably high concentrations.

The solvent used in the extraction markedly influenced the amino acid profiles. Aqueous extracts contained the highest levels of arginine, histidine, and lysine across all plant parts. The extracts prepared with 40% aqueous ethanol yielded the highest concentrations of aspartic and glutamic acids, whereas those prepared with 70% aqueous ethanol yielded the highest levels of leucine and proline. These solvent-dependent differences are consistent with the structural and polarity characteristics of the amino acids.

Previous studies [11] have reported histidine as the predominant amino acid in the leaves. However, we found that isoleucine was the most abundant amino acid in the leaf extracts. In previous studies, glutamic acid was reported to be the major amino acid in the aerial parts of *S. officinale*. In our study, glutamic acid was likewise present at relatively high levels in the 40% aqueous ethanol extracts of leaves and flowers, although this pattern did not hold consistently across all aerial-part extracts. Methionine and arginine have previously been reported as dominant amino acids in the roots [11], whereas we found that histidine and lysine were the most abundant in root extracts. In addition, we detected several amino acids, to our best knowledge, reported for the first time in *S. officinale* in the scientific literature. These amino acids include alanine, aspartic acid, leucine, phenylalanine, proline, serine, threonine, and valine.

#### 2.1.3. Pyrrolizidine Alkaloids

A total of eight PAs were detected in the quantitative analysis. The levels of four pyrrolizidine alkaloids, namely intermedine and lycopsamine and their respective N-oxide forms, were determined. Four other PAs (7-O-acetylintermedine, 7-O-acetyllycopsamine and their corresponding N-oxide forms) were also tentatively identified. These findings are analogous to those previously reported [24]. The present compounds were intermedine, lycopsamine, intermedine N-oxide, and lycopsamine N-oxide. The content of these compounds in the *S. officinale* extracts is presented in Table 1.

The remaining four PAs showed retention profile and product ions consistent with the acetylated derivatives of intermedine and lycopsamine (7-O-acetylintermedine, 7-O-acetyllycopsamine and their corresponding N-oxide forms). The fragments of 7-O-acetylintermedine and 7-O-acetyllycopsamine were observed at their specific precursor ions of 342 *m*/*z*. The presence of product ions at 180 *m*/*z* and 120 *m*/*z* is indicative of PAs. Previously, Trifan et al. [18] revealed the 120 *m*/*z* product ion of various types of PAs in *S. officinale* roots. According to Stanoeva et al. [25], the 180 *m*/*z* product ion is considered characteristic of open-chain diesters of PAs. This ion was detected in the spectra of 7-O-acetylintermedine and 7-O-acetyllycopsamine, which were identified in *S. officinale* roots [18]. 7-O-Acetylintermedine-N-oxide and 7-O-acetyllycopsamine-N-oxide were observed at their corresponding [M+H]^+^ *m*/*z* value of 358. The presence of specific product ions at 180 *m*/*z* and 214 *m*/*z* was also detected for these compounds, consistent with previously published patterns for such PAs [18,25]. However, in the absence of authentic reference standards for 7-O-acetylintermedine, 7-O-acetyllycopsamine and their N-oxides, the compounds could not be quantified with full confidence.

The lowest concentrations of total quantified PAs were observed in leaf extracts, with a maximum of 26.32 µg/g in the S7 (40% ethanol) sample, which yielded 20.38 µg/g of dominant lycopsamine N-oxide (Table 1). In comparison, the sum content of alkaloids in the root extracts was in the range between 332.80 µg/g (S1) and 639.01 µg/g (S3), while the content of alkaloids in the extracts derived from flowers was found to vary from 185.80 µg/g (S11) to 743.03 µg/g (S13). This may be because PAs are biosynthesised in roots and young leaves, where they are transported to flowers. The leaves used in this study were from a blooming plant. In previous studies, the alkaloid content of root samples has been shown to exceed that of dried leaves. Roeder et al. (1995) reported that, based on the findings of a limited number of studies, dried leaves exhibit 1.6–12.5 times lower quantities of PAs (0.02 to 0.18 of leaves and 0.25 to 0.29% of roots) [26]. Kimel et al. [27] found that the sum of PAs present in the leaves of *S. officinale* from Polish sources was 5.5 to 13.7 times lower than that found in roots (0.11 to 0.24 mg/g for leaves and 0.60 to 3.28 mg/g for roots, respectively). The total PA concentration in the flowers has been previously reported as 243 µg/g in dry weight and 41 µg/g in fresh weight [17]. In our study, the content of individual PAs in the flower extracts ranged from 11.09 to 74.14 µg/g for intermedine and from 12.75 to 111.50 µg/g for lycopsamine, while their respective N-oxide forms ranged from 0 to 298.41 µg/g and from 0.16 to 389.67 µg/g.

The PA content in plant material varies in growing regions and storage time, dropping already within a month of storage [22]. The solvent used in the extraction may affect the extract content. In the present study, we found that the water extracts contain higher amounts of non-N-oxidised forms of PAs (lycopsamine and intermedide). The lower content of the whole sum of quantified alkaloids (332.80 µg/g of non-N-oxidised forms of root extract, 8.70 µg/g of leaf extract, and 185.80 µg/g of flower extract, in contrast to the findings of 536.24–639.01, 2.43–26.32, and 552.39–743.03 µg/g of sum of PAs, respectively). Such specificity of the extraction solvent can be purposefully used to develop preparations with a targeted PA composition or reduced total PA content. It is important to note that N-oxides are generally less toxic than their respective PAs, provided they are not reduced back to PAs. When administered orally, the toxicity of N-oxide derivatives may be comparable to that of non-N-oxidised forms of PAs. However, when administered topically, N-oxide derivatives exhibit better tolerability due to their physicochemical properties and their minimal skin penetration [28,29].

The root and flower extracts exhibited broadly similar PA profiles. Among these extracts, intermedine-N-oxide and lycopsamine-N-oxide were present at the highest concentrations, followed by their corresponding unoxidized forms. Intermedine and lycopsamine were detected at several-fold higher levels in the flower water extracts compared with the root extracts prepared using the same solvent. Notably, in the aqueous flower extracts, no N-oxide forms were detected. By contrast, the leaf extracts contained markedly lower PA concentrations, with a maximum level of 0.21 µg/g—substantially lower than detected in both root and flower extracts for the same Pas (>3.1 µg/g).

In the aqueous extracts, intermedine and lycopsamine were the predominant Pas. Their concentrations, however, decreased progressively with increasing ethanol content in the extraction solvent. In contrast, their N-oxide derivatives showed the opposite trend, reaching their highest levels in the 70% aqueous ethanol extracts and decreasing as the water proportion increased.

Trifan and co-workers reported 0.04 mg/g intermedine, 0.03 mg/g lycopsamine, and 1.23 mg/g and 0.94 mg/g of their respective N-oxides in the raw root extracts prepared by 65% ethanol as a solvent system [24]. In comparison, the concentrations observed in our study were substantially lower.

Overall, the leaf extracts contained the lowest PA levels (not exceeding 0.21 µg/g). This is consistent with the established understanding that PA biosynthesis occurs primarily in roots and young leaves, and that the compounds are then translocated to the flowers. The leaves analyzed in this study were collected at full bloom, which may explain their reduced PA content. Flowers have been previously reported to contain 2.075 ± 0.132 µg/g PAs in cryo-lyophilised samples [17]. In our study, individual PA concentrations in flowers ranged from 0.24 to 5.97 µg/g. The PA levels in plant material are known to vary depending on the growing region and storage (with significant declines occurring within the first month of storage) [22]. Therefore, the discrepancies between our findings and previously published data may be attributed to environmental growing conditions, extraction methodology, and the time elapsed between harvesting and extraction.

### 2.2. Pharmacological Research on Hemostatic and Wound-Healing Activity and Cytology of Wound Exudate

#### 2.2.1. Hemostatic Activity

**Figure 1:**
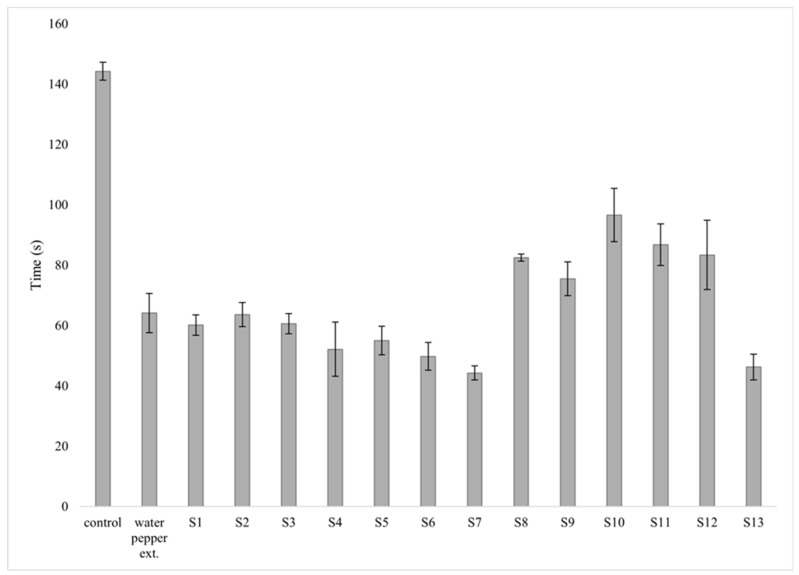
Hemostatic activity of 1% *S. officinale* extracts. Bleeding cessation time (mean ± SD).

The hemostatic activity of extracts derived from *S. officinale* raw plant material was studied in rats by measuring bleeding cessation time after a standardised skin incision. The effects of the studied extracts were compared with those of the control and reference groups treated with a standard hemostatic agent (Water pepper (*Polygonum hydropiperis*) extract). The study demonstrated that the reference preparation significantly reduced bleeding time in rats (64.1 s vs. 144.2 s with the control group) (Figure 1). The most effective extract was S7, which achieved complete cessation of bleeding within 44.2 s, surpassing even the reference preparation. The extracts S6 and S13 also showed a notable hemostatic activity, terminating bleeding faster than the reference preparation. The corresponding values for the extracts S6 and S13 were 49.7 and 46.2 s, respectively.

Although *S. officinale* has been widely used to promote wound healing and bone regeneration, its hemostatic activity has not been systematically studied to date. To the best of our knowledge, this is the first study to experimentally confirm and quantitatively evaluate the hemostatic activity of *S. officinale*. Traditionally, the hemostatic effect of *S. officinale* has been attributed solely to its astringent action, specifically that of tannins. However, our study suggests a more complex mechanism. The synergistic interaction of polyphenolic compounds and a specific set of amino acids (particularly proline and glycine) may enhance vascular response and activate local coagulation factors.

#### 2.2.2. Wound-Healing Activity

**Figure 2:**
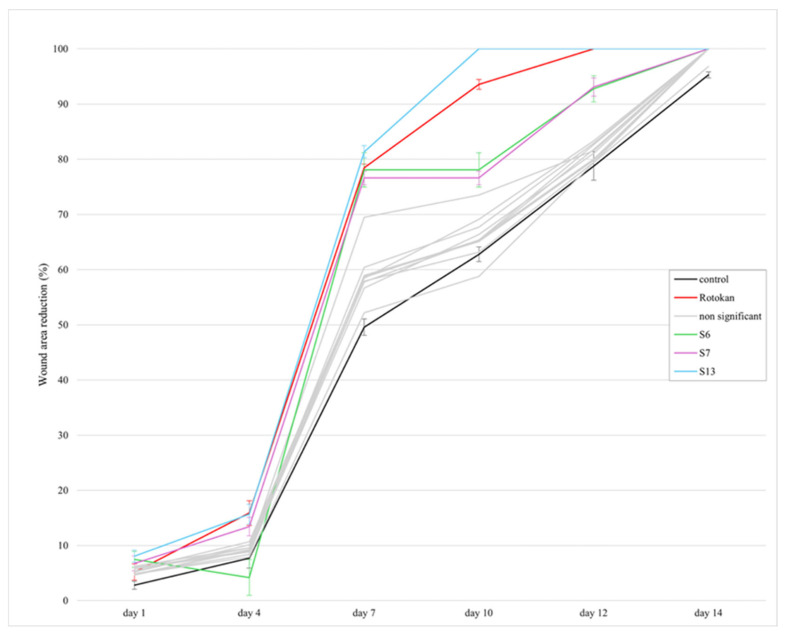
Most promising wound healing activity of 1% *S. officinale* extracts (mean wound area reduction ± SD).

The wound-healing activity of extracts from *S. officinale* was assessed in rats by measuring wound area after a standardised skin incision. The effects of the extracts were compared with those of the control and reference groups treated with a standard wound-healing preparation (“*Rotokan*”). The reference preparation “*Rotokan*” significantly reduced the wound area already on the first day (5.2%). In the control group, the reduction in wound area was 2.8% (Figure 2).

As seen in Figure 2, the greatest increase in wound-healing percentage occurred between days 4 and 7 (from 15.9% to 59.6%). The wound was completely closed on day 12 in the rats treated with “*Rotokan*”. The most effective extract was S13, which achieved complete wound healing by day 10 (faster than observed even with the reference preparation). The S13 extract also showed the most pronounced wound-healing dynamics between days 4 and 7 (similar to “*Rotokan*”). The extracts S6 and S7 likewise exhibited notable wound-healing activity throughout the experiment, and the wound-healing activity was significantly better than that observed in the control group. On day 12, the wound-healing percentages for the extracts S6 and S7 were 92.8% and 93.1%, respectively.

We found that the extracts S6, S7, and S13 exhibited significant biological activity, and moreover, S13 showed the highest wound-healing potential. These effects exceeded those reported in earlier comparable studies using leaf or root extracts prepared with different solvents and concentration levels (3–20%) [7,8]. Our results indicate superior performance of the extracts compared to those reported by Araújo et al. [8], who investigated leaf extracts of *S. officinale* prepared with a water–propylene glycol mixture (60:40) as the solvent system, with a 48 h maceration. Araújo et al. [8] reported reduced inflammation and faster progression through the proliferation phase (the total wound-healing time, however, was not specified). Another study [7] evaluated wound healing in rats using the root extracts of *S. officinale* (extracted with 65% ethanol under reflux conditions). The authors reported complete wound healing by day 12 in rats treated with a 20% cream compounded from the root extract. In our study, the S13 extract achieved complete wound healing by day 10, with a similar cytological pattern of healing (including intensive granulation and re-epithelialization). Since the present extract S13 shows marked efficacy in wound healing even at a concentration of only 1%, it would be feasible to develop cost-effective extract-based topical formulations for wound treatment, thus advancing beyond the current clinical protocols that require prolonged application. At the same time, the investigation should be extended to include also the models of chronic and infected wounds to confirm the universality of wound healing mechanisms, and to evaluate potential risks of hyperplasia or excessive scarring during long-term use.

#### 2.2.3. Cytology of Wound Exudate

Cytological evaluation of wound imprints on days 1, 3, and 7 showed clear quantitative differences between the control group, reference group (“*Rotokan*”), and the rats treated with the experimental extracts (Table 2).

In the control group, neutrophils dominated on day 1 (median 95%). By day 3, the percentage frequency of neutrophils decreased to 80%, while the frequency of macrophages increased to 15%. Fibroblasts were present at low frequency (5%). On day 7, neutrophils remained at 58% frequency, while fibroblast frequency increased to 15%. In the “*Rotokan*” group, neutrophils measured 88% on day 1, and the frequency level was decreased to 60% by day 3. The percentage frequency of macrophages increased to 22%, and the frequency of fibroblasts reached 3% on day 3. On day 7, the frequency of neutrophils and fibroblasts was 30% and 37%, respectively. Among the extracts, S13 showed the largest reduction in neutrophils on day 3 (50%) compared with the “*Rotokan*” group (60%) and the control group (80%). In addition, the use of extract S13 resulted in increased macrophage counts during days 1–3 and fibroblast levels of 37% on day 7. Extracts S6 and S7 showed consistently higher fibroblast and lower neutrophil counts than the control group throughout the study.

**Figure 3:**
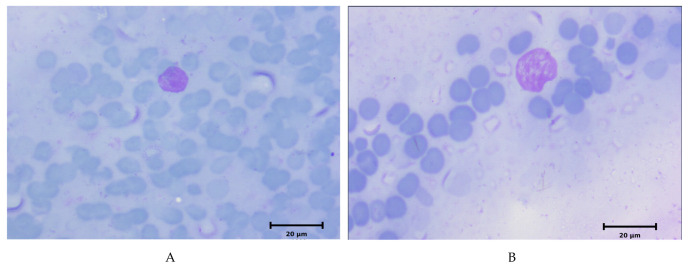
Cytological characteristics of the cellular composition of wound exudate, group S13, microscope magnification ×1000. (**A**) The 3rd day. The microphotograph shows predominance of cellular debris and erythrocytes, against which a single large macrophage-like cell is visible. The macrophage has a rounded shape, well-defined basophilic cytoplasm, and a relatively uniform, non-segmented nucleus. No signs of active neutrophilic infiltration (numerous segmented forms) are observed. Neutrophils are represented by single cells showing morphological features of reduced functional activity, consistent with the decline of the acute inflammatory reaction. Destructive forms and massive leukocyte degradation are absent. (**B**) The 7th day. The microphotograph demonstrates a marked reduction in inflammatory cells. Neutrophils appear only as isolated cells or are absent from the field of view. No signs of active neutrophilic degranulation, leukocytic debris, or tissue destruction are present. A macrophage line cell with decreased cytoplasmic activity is visible, which is characteristic of the later stages of repair. The background is dominated by cellular elements, including fibroblastic and fibrocytic components, which form the basis of granulation tissue. Blood elements are present in minimal amounts and show no signs of stasis or changes in hemorrhage.

Cytological evaluation showed that extract S13 caused a more pronounced shift in cellular composition than the reference preparation “*Rotokan*” (Figure 3). On day 3, neutrophils in the S13 group decreased to 50% (45–54), which was significantly lower than in the “*Rotokan*” group (60%) (*p* < 0.05). At the same time, macrophage levels increased to 17% (13–20), while lymphocyte levels remained stable. On day 7, the S13 group showed the lowest neutrophil infiltration at 28% (21–33) and the highest fibroblast proportion at 37% (26–43). Both values exceeded the corresponding values found with the control and *Rotokan* groups. All values are presented as medians (min-max), and intergroup comparisons were performed using the Mann–Whitney U-test (*p* < 0.05).

Cytological monitoring of wound exudate revealed the mechanism underlying this acceleration. The study clearly demonstrated a reduction in the inflammatory phase and a positive effect on all its stages. Under the influence of S13, a rapid decrease in neutrophil levels was observed as early as day 3, thereby reducing the intensity of secondary alteration. This is critically important, as excessive neutrophil infiltration leads to the release of proteolytic enzymes and secondary tissue damage. The treatment was associated with increased macrophage counts during the early stages of healing.

An elevated macrophage population in the early stages of inflammation promotes more rapid wound decontamination and initiates the transition to the final stage—proliferation, which is realised, among other mechanisms, through effective fibroblastogenesis. In our study, we demonstrated that by day 7, the proportion of fibroblasts in the S13 group was significantly higher than in the “*Rotokan*” group, which correlated with accelerated granulation tissue formation and an earlier onset of epithelialization. It should be noted that the observed effects are based on the biochemical determinants of *S. officinale* extract activity and are directly correlated with its phytochemical profile. For the most effective extract, S13, RA may have contributed to the observed effects, given its reported antioxidant and anti-inflammatory properties [30,31]. However, the present study does not permit direct attribution of the cellular responses to any individual phytochemical constituent.

Similarly, caffeic acid and isoquercitrin have been associated with regenerative and anti-inflammatory effects, which support tissue repair processes [32]. An additional positive factor is the presence of amino acids. In particular, proline and glycine serve as essential substrates for collagen synthesis by fibroblasts, while lysine contributes to the formation of intermolecular cross-links within the dermal matrix structure [33]. The presence of other amino acids, such as leucine, lysine and glycine, further supports enhanced regenerative capacity. The cytological findings indicate an accelerated transition from the inflammatory phase to the proliferative phase of wound healing in the S13-treated group.

The accelerated progression of wound-healing phases observed in our study, including the rapid reduction in neutrophils and increased fibroblast activity, suggests enhanced tissue regeneration dynamics.

Overall, the observed pharmacological effects are likely due to synergistic interactions among multiple bioactive constituents. This synergism may explain the enhanced efficacy of the extracts compared to known analogues, including water pepper extract and “*Rotokan*”. The present findings confirm the traditional use of *S. officinale* in wound healing and extend its pharmacological relevance by demonstrating improved activity through optimised extraction and compositional targeting.

#### 2.2.4. Linking Phytochemical Composition to Wound-Healing and Other Activities

The superior wound-healing and hemostatic activity of *S. officinale* leaf (S7) and flower (S13) extracts appears to be associated with their distinct phytochemical profiles, particularly their high content of phenolic compounds and amino acids, as well as organ-specific differences in PAs. Both extracts were rich in phenolic compounds, especially RA, caffeic acid and flavonoids, which are known for their antioxidant and anti-inflammatory properties that promote tissue repair. These effects may reduce oxidative stress and modulate inflammatory responses at the wound site, thus facilitating regeneration. RA has been shown to enhance collagen deposition and accelerate wound healing [34,35]. Overall, phenolic compounds contribute to wound healing through antioxidant, anti-inflammatory and antimicrobial mechanisms [36], which is consistent with the superior in vivo performance of S7 and S13.

In addition, the high content of amino acids, particularly proline, glutamic acid and aspartic acid, may support collagen biosynthesis and cellular metabolism during wound healing. Amino acids are known to promote tissue repair by supporting cell proliferation and protein synthesis [37,38,39].

Beyond wound closure, the observed hemostatic activity may also be linked to the phytochemical composition of the extracts. Phenolic compounds, particularly flavonoids, are known to modulate haemostasis through effects on vascular function, platelet activity and coagulation pathways [40]. In addition, phenolic constituents such as RA and caffeic acid exhibit antioxidant and anti-inflammatory activities [34], which may further improve wound conditions.

From a safety perspective, the comparatively low PA content in *S. officinale* leaf extract S7 suggests a more favourable balance between efficacy and safety. Overall, the enhanced activity of S7 and S13 is likely due to the combined effects of phenolic compounds and amino acids, as well as organ-specific differences in PA content. Importantly, although the biological activity of Symphytum species has traditionally been attributed mainly to PAs, the present findings indicate that other constituents, particularly phenolic compounds and amino acids, also play a significant role in the pharmacological effects, suggesting a multi-component mechanism of action.

These results support the traditional use of *S. officinale* in wound treatment and suggest that aerial parts may represent important contributors to its biological activity, emphasizing the relevance of organ-specific composition in understanding its pharmacological effects.

To further explore possible relationships between phytochemical composition and biological activity, Spearman correlation analyses were performed between the major quantified phenolic parameters (total phenolic compounds, hydroxycinnamic acids, flavonoids, and rosmarinic acid content) and both hemostatic and wound-healing activities. No statistically significant correlations were observed (*p* > 0.05). The strongest associations were observed between flavonoid content and wound-healing activity (r = 0.55, *p* = 0.083) and between rosmarinic acid content and wound-healing activity (r = 0.47, *p* = 0.15), although neither reached statistical significance. These findings suggest that the observed biological effects cannot be readily attributed to a single phytochemical class and are more likely associated with the combined contribution of multiple constituents present in the extracts.

Limitations of the study. The present study has several limitations. The pharmacological evaluation was performed using a single extract concentration (1%), and dose–response relationships were therefore not assessed. In addition, although significant wound-healing and hemostatic activities were observed, the underlying molecular mechanisms and comprehensive toxicological aspects require further investigation. Future studies should include dose-dependent experiments, mechanistic validation, and safety assessment of the extracts.

Although the phytochemical composition of the most active extracts suggests that phenolic compounds and amino acids may contribute to the observed biological effects, the present study was not designed to identify molecular targets, establish direct structure–activity relationships, or investigate the molecular and biochemical mechanisms underlying wound healing. Therefore, the proposed mechanisms are based on cytological observations and published literature rather than direct mechanistic evidence. Future studies should investigate inflammatory mediators, growth factors, and tissue-remodelling pathways, and may also employ bioactivity-guided fractionation, target-based assays, and in silico approaches to identify the constituents responsible for the observed activities and to clarify their mechanisms of action.

## 3. Materials and Methods

### 3.1. Plant Material

*Symphytum officinale* flowers, leaves and roots (up to 200 g of raw material) were collected in the wild in Luunja River Port, Tartu County, Estonia (coordinates 58°21′16″ N, 26°53′05″ E). The plant material was botanically identified by Prof. Ain Raal (Institute of Pharmacy, University of Tartu, Estonia), and a voucher specimen (Bor/Soff1) was deposited at the Institute of Pharmacy, University of Tartu. The plant material was dried for ten days at room temperature, away from direct sunlight. Before analysis, the plant material was stored in paper bags.

### 3.2. Preparation of Extracts

The extraction procedure was performed as previously described [41,42], with minor modifications. The dried *S. officinale* roots (50.0 g), leaves (20.0 g) and flowers (9.0 g) were extracted with 40% and 70% aqueous ethanol solutions (respectively 250 mL, 200 mL and 100 mL) by macerating at room temperature for 24 h. The extraction procedure was repeated twice, using the same solvent volumes at each stage. The resulting liquid extracts were combined and filtered. Aqueous extracts were prepared from 20.0 g of dried flowers, 20.0 g of leaves, and 30.0 g of roots of *S. officinale* as infusions at a ratio of 1:10 (200 and 300 mL of water, respectively). The mixtures were heated to 100 °C for 15 min (flowers and leaves) and 30 min (roots), and then macerated for 24 h. The extracts were then filtered.

The polysaccharide and polyphenolic fractions of the aqueous extracts were also separated. For this purpose, 50.0 g of dried *S. officinale* leaves and 100.0 g of roots were extracted using the same procedure described above. The extracts were then filtered and concentrated. Polysaccharides were precipitated with three volumes of 96% ethanol and subsequently separated by filtration [43]. The resulting polysaccharide precipitates were washed with ethanol and acetone and dried by lyophilisation. The supernatants were then concentrated and dried to obtain dry extracts.

All extracts were concentrated separately using a Buchi B-300 rotary vacuum evaporator (Buchi AG, Flawil, Switzerland) under the following conditions: vacuum 150 mbar, rotation speed 50 rpm, and heating bath temperature 85 °C. The viscous concentrates were subsequently lyophilised using a SCANVAC COOLSAFE 55-4 Pro (LaboGene ApS, Lillerød, Denmark) apparatus.

The dry extracts were designated as S1–S13, and their descriptions are provided in Table 3. These designations were used throughout the study.

### 3.3. Spectrophotometric Assay of Main Groups of Phytochemicals

The quantification of key phenolic constituents (hydroxycinnamic acids, flavonoids, and total phenolic compounds) in the dry extracts of *S. officinale* roots, leaves, and flowers was carried out using a Shimadzu UV—1800 spectrophotometer (Shimadzu Corporation, Kyoto, Japan) following the procedures described in the European Pharmacopoeia [44].

Hydroxycinnamic acids were determined via their reaction with sodium molybdate and sodium nitrite, using chlorogenic acid as a calibration standard. Optical density was measured at 525 nm [41,45]. The flavonoid content was assessed by complex formation with aluminium chloride, with absorbance recorded at 417 nm; rutin served as the reference compound [42,46]. Total phenolic content was measured at 270 nm employing gallic acid as the standard [45,47]. All analyses were performed in triplicate to ensure statistical reliability.

### 3.4. Assay of Polyphenolic Compounds by UPLC-MS/MS

The polyphenolic composition of *S. officinale* dry extracts was quantified using a UPLC–MS/MS analytical platform. The separation of polyphenolic compounds from the test sample solution was conducted in accordance with a slightly modified experimental procedure that had been published previously [48]. The experimental procedure was conducted using an Acquity H-class UPLC system (Waters, Milford, MA, USA) equipped with a YMC Triart C18 column (100 × 2.0 mm, 1.9 μm). The column temperature was maintained at 40 °C. The mobile phase consisted of 0.1% formic acid in water (eluent A) and MS-grade acetonitrile (eluent B). The mobile-phase delivery rate was 0.5 mL/min. The chromatographic gradient was programmed as follows: 0–1 min, 95% A; 1–5 min, linear increase in B to 30%; 5–7 min, increase to 50% B; 7.5–8 min, column wash at 100% B, followed by re-equilibration to the initial conditions, for a total run time of 10 min.

Mass spectrometric detection was performed using an Xevo TQD triple-quadrupole tandem mass spectrometer (Waters, USA) operating in negative electrospray ionisation (ESI) mode. The capillary voltage was set to −2 kV, and the cone voltage to 30 V. The desolvation gas (nitrogen) was heated to 400 °C and supplied at 700 L/h. The curtain gas flow was maintained at 20 L/h. The ion source temperature was kept at 150 °C. Compound identities were confirmed by comparison with authentic analytical standards based on retention time and characteristic MRM transitions. This was performed by means of data analysis with MassLynx v4.1 software. Quantification was performed on the TargetLynx XS package (MassLynx 4.1 software package) using linear regression calibration and standard dilution approaches. Representative MRM chromatograms of quantified polyphenolic compounds in the S9 sample and reference standards are presented in Supplementary Figures S1 and S2 (Supplementary Materials).

### 3.5. Assay of Amino Acids by UPLC-MS/MS

The analysis of amino acids in *S. officinale* dry extracts was performed using an Acquity H-class UPLC system (Waters, Milford, MA, USA) coupled to an Xevo TQD mass spectrometer (Waters, Milford, MA, USA). The procedure was conducted under conditions analogous to those previously published [49]. A 1 µL aliquot of each extract was injected onto a BEH Amide column (150 mm × 2.1 mm, 1.7 µm; Waters), maintained at 25 °C. The mobile phase consisted of 10 mmol ammonium formate with 0.125% formic acid in water (eluent A) and acetonitrile (eluent B), delivered at 0.6 mL/min. The gradient program was applied as follows: 0–1 min, 95% B; 1–3.9 min, decrease to 70% B; 3.9–5.1 min, decrease to 30% B; 5.1–6.4 min, column wash at 70% A; followed by re-equilibration to the initial conditions at 6.5 min, resulting in a total analysis time of 10 min.

Detection was carried out in positive ESI mode with a capillary voltage of +3.5 kV and a cone voltage of 30 V. The desolvation gas flow was set at 800 L/h and heated to 400 °C, while the ion source temperature was maintained at 120 °C. The identification and quantification of amino acids was performed in the same manner as for polyphenolic compounds. The MRM chromatograms of the quantified amino acids in the test solution of the S12 sample and the reference solution are presented in Figures S3 and S4 (Supplementary Materials).

### 3.6. Assay of Pyrrolizidine Alkaloids by UPLC-MS/MS

Analysis of PAs was performed on the same UPLC-MS/MS system as for polyphenolic compounds and amino acids. The analytical procedure was based on a modification of the conditions of a previously reported method [50]. Chromatographic separation was done on a Waters ACQUITY BEH C18 column (100 mm × 2.1 mm, 1.7 µm). The injection volume was set at 2 µL, and the column was maintained at 30 °C. The mobile phase was delivered at 0.3 mL/min. Mobile phases A (0.1% formic acid with 5 mmol ammonium acetate in water) and B (methanol) were supplied using a gradient method with the following settings: initial conditions of 95% A were held for 0.5 min. At 2 min, 90% of A was set. 85% of A was reached at 4 min. 80% of A was attained at 10 min. 50% of A was set at 14 min. Column flush with 10% A for 16 min. Initial conditions were returned at 16.1 min for a total run time of 18 min.

Mass spectrometric analysis was performed using positive electrospray ionisation (ESI+) with a capillary voltage of +3.5 kV and a cone voltage of 30 V. The desolvation gas was delivered at a flow rate of 650 L/h and heated to 500 °C, while the ion source temperature was maintained at 120 °C. The identification and quantification of PAs were performed in the same manner as for polyphenolic compounds. The retention times of the peaks in MRM chromatograms of test solutions were compared with those of analytical-grade reference standards to identify quantified PAs (Figures S5–S7 of Supplementary Materials). Quantification was performed using the calibration curves generated by linear regression (per the standard dilution method).

### 3.7. Pharmacological Study

The pharmacological studies of *S. officinale* extracts were conducted at the Educational and Scientific Medical Laboratory Centre, with a vivarium at the Zaporizhzhia State Medical and Pharmaceutical University (ZSMPU), Ukraine. Extracts of series S1–S13 (Table 3), prepared from *S. officinale*, were stored in a dry, light-protected place at room temperature and 40–60% relative humidity in hermetically sealed vials. Before conducting the experiments, 1% aqueous solutions of the extracts were prepared; to ensure complete dissolution, the mixtures were heated to 40 °C. No excipients or stabilisers were used.

The experiments were carried out in accordance with international and national standards for the humane treatment of animals: the Law of Ukraine No. 3447-IV “On the Protection of Animals from Cruelty” [51], the “European Convention for the Protection of Vertebrate Animals Used for Experimental and Other Scientific Purposes” [52]. The research was approved by the Bioethics Commission of the ZSMPU (protocol 4 dated 12 March 2026). The study was conducted on white Wistar rats weighing 190–220 g. The animals were kept under standard vivarium conditions with free access to water and standard feed [53].

#### 3.7.1. Hemostatic Activity

Hemostatic activity was evaluated by measuring the time required for complete cessation of bleeding after making a skin incision on the laboratory animals. The incision was performed under local anaesthesia (subcutaneous administration of sodium novocaine at a dose of 60 mg/kg body weight) to avoid pain and stress in the animals. One day before the experiment, the hair on the animals’ backs (along the spine) was carefully shaved, and the skin was treated with an antiseptic (70% ethanol). Under aseptic conditions, an incision was made in the depilated area of the back, followed by application of the tested extracts onto the wound for 30 s. If bleeding did not stop within this period, the extract was reapplied every 15 s for an additional 5 s [54,55].

The animals were divided into groups (8 animals in each). The control group (pathology) comprised the animals with a wound that received no treatment. The experimental groups S1–S13 comprised the animals whose wounds were treated daily with the tested extracts S1–S13 (applied topically). The positive control group (reference group) comprised animals treated with a standard preparation with established hemostatic activity (“*Water pepper extract*” (series no. 20924, “*Ternopharm*”, Ukraine)—liquid extract from the *Persicaria hydropiper* herb). For each group, the time required for complete cessation of bleeding was measured. Statistical analysis and comparison of the results were performed using one-way ANOVA.

#### 3.7.2. Wound-Healing Activity

Wound-healing activity was evaluated by performing a skin incision on the backs of the laboratory animals.

Skin incisions were performed in accordance with the procedure described in Section 3.7.1. Under aseptic conditions, a longitudinal linear incision was made in the depilated area, involving the skin and subcutaneous tissue. The wound size was 10 × 15 mm (average area 150 mm^2^).

The animals were divided into a total of 15 groups (8 animals in each). The control group (pathology) comprised the animals with a wound that received no treatment. The experimental groups S1–S13 comprised the animals whose wounds were treated daily with the experimental extracts S1–S13 (topical applications). The positive control group (reference group) comprised animals treated with a standard drug product with proven wound-healing properties (“Rotokan”). The standard drug preparation used was “*Rotokan*” (batch no. 160625, “*Lubnypharm*”, Ukraine), a mixture of tinctures derived from the flowers of *Matricaria chamomilla* and *Calendula officinalis*, and from the herb *Achillea millefolium*. The experimental extracts and reference preparation were applied onto the wound on a daily basis, followed by the measurement of the wound area. For each group, the following two parameters were calculated from the data: (1) wound area in mm^2^ (indicating the dynamics of size reduction) and (2) healing rate (percentage reduction relative to the initial area). The results obtained with the “*Rotokan*” group and control group were compared using one-way ANOVA. After zero point, wound healing was assessed in the rats on days 1, 4, 7, 10, 12, and 14. The results are expressed as the mean percentage of wound closure (M) accompanied by the corresponding standard deviation (SD) [55,56,57].

#### 3.7.3. Cytology of Wound Exudate

An additional criterion for assessing wound-healing activity was the cytological analysis of imprint smears obtained from the wound surface during the experiment. The wound-healing process was monitored using imprint smears taken from the wound surface according to the method of M. V. Pokrovska and M. S. Makarov, and modified by D. M. Shteinberg [54]. The samples were collected on days 1, 3 and 7 of the wound-healing process. For cytological examination, the imprint preparations were prepared using standard glass slides. Clean slides were stored in ethanol in a tightly closed container. Before use, a slide was removed with sterile forceps, flamed over an ethanol burner to disinfect, and warmed to obtain an imprint of the wound surface. After imprinting, the preparations were fixed in methanol for 5 min and stained using the Romanowsky-Giemsa method. The stained smears were examined with an Axio Star Plus microscope (Carl Zeiss AG, Jena, Germany). Within each field of view, the following cellular components were quantified: neutrophils (including dystrophic forms), phagocytic activity, eosinophils, lymphocytes, monocytes, multinucleated giant cells, macrophages, fibroblasts, endothelial and epithelial cells, and fibrin fibres [58,59,60].

### 3.8. Statistical Analysis

To evaluate statistical significance, Student’s *t*-test was applied with a threshold of *p* ≤ 0.05. For assays involving multiple groups, appropriate multiple-comparison methods were used when necessary. These methods include one-way ANOVA followed by post hoc tests (e.g., Tukey’s test). All statistical analyses were performed in Microsoft Excel 2019 (Microsoft Corporation, Redmond, WA, USA), and in accordance with the requirements of the State Pharmacopoeia of Ukraine [61]. Exploratory Spearman rank correlation analyses were additionally conducted to evaluate potential associations between phytochemical composition and biological activity.

## 4. Conclusions

In the present study, a comprehensive phytochemical and pharmacological evaluation of the *S. officinale* extracts obtained from roots, leaves and flowers was performed. A total of 12 phenolic compounds, 14 amino acids, and 8 PAs were identified, and a pronounced organ-specific chemical profile was demonstrated. The extracts prepared from leaves and flowers presented the highest diversity of phenolic compounds. RA, caffeic acid, and isoquercitrin were identified as the dominant constituents. The extracts prepared from roots showed comparatively lower phenolic diversity but higher PA content. Amino acid profiling revealed that extracts prepared from flowers had the highest total abundance of proline, aspartic acid, and glutamic acid, which are known to be involved in tissue regeneration. The solvent system used in extraction significantly influenced the phytochemical composition.

Pharmacological studies showed that the extracts prepared from *S. officinale* leaves (S7: 40% ethanol) and flowers (S13: 70% ethanol) possess pronounced hemostatic and wound-healing activity in rats. The use of extract S13 resulted in complete wound closure by day 10, while S7 significantly reduced bleeding time.

These effects may be associated with the combined contribution of polyphenols, amino acids, and other constituents present in the extracts; however, the present study did not establish direct relationships between individual phytochemicals and the observed biological activities. Importantly, these findings provide experimental support for the traditional use of *S. officinale* in wound treatment and suggest that the plant’s aerial parts may contribute more significantly to its therapeutic activity than is commonly assumed. Our results indicate that the present extracts could be promising candidates for the development of novel hemostatic and wound-healing preparations.

The findings of this study suggest that the biological activity of *S. officinale* reflects a multi-component system involving phenolic compounds, amino acids and PAs, rather than being attributable to a single class of constituents.

Future studies should focus on elucidating the molecular mechanisms underlying the observed activities, identifying the constituents responsible for the biological effects, and evaluating the efficacy and safety of these extracts in advanced wound-healing models and formulation development.

## Figures and Tables

**Table 1:** Content of polyphenolic compounds, amino acids, and pyrrolizidine alkaloids in the *S. officinale* extracts.

**Substance**	**Retention Time, Min**	Content in the Extract, µg/g										
		S1	S2	S3	S4	S6	S7	S8	S9	S11	S12	S13
Neochlorogenic acid	3.03	0	0	0	0	0	74.93 ± 7.76	90.60 ± 3.88	106.01 ± 11.48	0	0	0
Isoquercitrin	5.44	0	0	0	0	434.37 ± 21.09	1734.12 ± 138.28	2136.84 ± 101.05	1300.24 ± 49.83	0	680.46 ± 56.31	1215.34 ± 68.00
Chlorogenic acid	3.91	0	0	0	0	0	0	0	109.40 ± 1.87	0	0	0
Phloridzin	6.02	0	0	0	0	0	0	0	32.51 ± 3.31	0	0	0
*p*-Coumaric acid	5.01	0	0	0	0	410.25 ± 25.22	0	0	188.28 ± 15.65	101.34 ± 7.03	0	0
Ferulic acid	5.35	0	0	0	0	90.60 ± 8.30	0	0	73.52 ± 3.63	33.48 ± 4.75	39.27 ± 2.94	59.15 ± 4.92
Isorhamnetin-3-glucoside	5.91	0	0	0	0	0	0	0	0	0	31.92 ± 4.32	59.65 ± 5.94
Caffeic acid	4.19	137.06 ± 9.91	0	42.25 ± 3.41	67.42 ± 5.15	1211.21 ± 38.79	584.47 ± 3.92	517.46 ± 22.18	872.54 ± 31.52	819.22 ± 9.60	642.39 ± 26.95	591.11 ± 14.77
3,4-Dihydroxybenzoic acid	2.54	0	0	0	0	0	0	0	219.09 ± 21.44	0	0	0
Kaempherol-3-O-glucoside	5.82	0	0	0	0	26.44 ± 7.39	144.10 ± 6.93	109.40 ± 2.29	81.69 ± 5.89	0	131.98 ± 6.87	203.71 ± 10.13
Hyperoside	5.38	0	0	0	0	38.37 ± 10.99	344.79 ± 31.25	343.55 ± 4.83	129.44 ± 4.17	0	149.03 ± 10.00	245.59 ± 27.95
Rosmarinic acid	6.06	1779.30 ± 144.81	1069.04 ± 51.52	4234.32 ± 157.26	3550.59 ± 106.33	5553.09 ± 357.51	11,397.33 ± 788.74	16,863.94 ± 987.32	4971.98 ± 158.92	7416.77 ± 237.41	8775.57 ± 522.01	17,632.96 ± 1742.68
Spectrophotometry, %												
Total polyphenolic compounds		2.85 ± 0.07	5.12 ± 0.22	5.34 ± 0.38	3.03 ± 0.11	7.48 ± 0.18	11.36 ± 0.88	11.46 ± 0.64	5.15 ± 0.04	10.3 ± 0.16	12.18 ± 0.37	10.40 ± 0.16
Hydroxycinnamic acids		0.50 ± 0.05	0.96 ± 0.10	0.97 ± 0.04	0.31 ± 0.01	1.28 ± 0.12	1.79 ± 0.05	2.31 ± 0.27	0.06 ± 0.05	1.64 ± 0.13	2.33 ± 0.11	1.50 ± 0.12
Flavonoids		0 *	0	0	0.27 ± 0.01	0 *	2.23 ± 0.48	4.67 ± 0.35	0.88 ± 0.06	0 *	2.06 ± 0.08	3.71 ± 0.19
Content of amino acids in the *S. officinale* extracts by UPLC—MS/MS												
Alanine		12.25 ± 0.26	7.14 ± 0.92	13.75 ± 0.93	10.07 ± 0.86	26.08 ± 1.31	28.64 ± 1.68	28.28 ± 1.54	63.27 ± 1.99	25.58 ± 1.86	51.86 ± 4.37	78.40 ± 2.10
Arginine		30.85 ± 0.29	28.81 ± 1.44	22.32 ± 1.43	33.42 ± 1.71	42.65 ± 0.84	41.61 ± 0.75	33.97 ± 1.36	45.22 ± 3.11	63.95 ± 3.07	50.20 ± 1.78	50.44 ± 2.25
Aspartic acid		0	0	0	0	28.66 ± 1.29	0	0	64.14 ± 1.17	46.88 ± 1.57	353.03 ± 11.56	153.04 ± 2.40
Glutamic acid		9.68 ± 0.16	12.39 ± 0.43	7.78 ± 0.31	10.07 ± 0.55	48.21 ± 1.77	157.11 ± 5.50	53.06 ± 2.55	115.17 ± 4.98	52.07 ± 0.28	213.14 ± 3.21	124.05 ± 2.86
Histidine		85.31 ± 2.76	82.40 ± 4.01	65.39 ± 0.52	96.83 ± 6.45	95.42 ± 4.17	75.85 ± 3.11	63.97 ± 2.48	62.39 ± 2.97	117.91 ± 2.77	76.47 ± 3.47	68.21 ± 1.99
Isoleucine		7.47 ± 1.75	3.45 ± 0.08	1.89 ± 0.55	8.06 ± 0.29	140.57 ± 1.81	143.76 ± 3.96	138.76 ± 7.26	101.87 ± 1.75	62.71 ± 1.31	62.20 ± 0.39	80.01 ± 3.32
Leucine		0	0	0	0	107.42 ± 6.34	93.32 ± 5.15	118.84 ± 7.05	101.98 ± 5.29	50.89 ± 2.39	42.67 ± 1.79	58.54 ± 0.40
Lysine		93.48 ± 4.00	88.48 ± 4.51	63.65 ± 3.59	85.00 ± 1.80	66.98 ± 4.14	37.99 ± 1.25	34.92 ± 3.49	35.62 ± 0.94	53.60 ± 2.64	45.57 ± 1.47	34.64 ± 0.16
Methionine		2.80 ± 0.21	1.81 ± 0.07	1.00 ± 0.07	1.62 ± 0.22	2.27 ± 0.45	3.55 ± 0.34	1.36 ± 0.07	0.81 ± 0.14	1.79 ± 0.10	1.64 ± 0.33	1.32 ± 0.20
Phenylalanine		4.64 ± 0.47	4.46 ± 0.94	3.45 ± 0.23	3.34 ± 0.40	28.36 ± 1.26	27.80 ± 0.62	25.64 ± 2.08	51.32 ± 2.72	16.64 ± 0.47	9.02 ± 0.24	13.89 ± 0.78
Proline		18.79 ± 1.25	27.13 ± 2.89	79.33 ± 3.21	83.37 ± 4.33	19.88 ± 1.58	21.85 ± 1.05	26.86 ± 3.17	20.91 ± 1.75	57.96 ± 3.79	76.53 ± 2.50	107.41 ± 5.94
Serine		14.40 ± 2.17	5.67 ± 1.45	9.59 ± 0.94	5.65 ± 0.45	47.35 ± 3.54	49.08 ± 1.56	43.17 ± 1.17	45.40 ± 0.65	46.71 ± 2.36	43.93 ± 2.45	54.75 ± 2.22
Threonine		0	0	0	0	40.87 ± 3.42	39.70 ± 3.85	39.33 ± 2.05	52.63 ± 3.00	46.47 ± 1.24	31.13 ± 1.40	61.50 ± 1.16
Valine		0	0	0	0	68.77 ± 4.08	80.35 ± 3.59	72.61 ± 0.28	145.05 ± 5.69	66.95 ± 1.23	74.64 ± 1.28	109.72 ± 7.06
Pyrrolizidine alkaloids												
Intermedine	6.11	20.52 ± 0.25	1.05 ± 0.10	7.18 ± 0.40	33.46 ± 1.73	1.27 ± 0.33	0.08 ± 0.07	0.12 ± 0.01	0.74 ± 0.18	74.14 ± 2.51	11.09 ± 0.29	23.20 ± 0.11
Lycopsamine	6.42	20.00 ± 3.08	0.89 ± 0.37	6.90 ± 0.35	37.86 ± 1.04	5.02 ± 0.63	0.42 ± 0.39	0.96 ± 0.23	1.25 ± 0.24	111.50 ± 3.89	12.75 ± 1.55	31.75 ± 1.18
Intermedine N-oxide	7.57	138.11 ± 9.73	257.38 ± 6.47	279.55 ± 4.19	200.42 ± 1.32	1.42 ± 1.72	5.44 ± 2.39	0.04 ± 0.06	1.76 ± 0.22	0	224.54 ± 4.66	298.41 ± 4.71
Lycopsamine N-oxide	8.01	154.17 ± 7.29	296.92 ± 3.01	346.38 ± 10.53	232.69 ± 4.81	1.00 ± 1.73	20.38 ± 2.44	1.31 ± 0.10	2.39 ± 0.56	0.16 ± 0.14	304.01 ± 7.86	389.67 ± 7.65

* *p* < 0.05.

**Table 2:** Cytogram of wound healing under the influence of the experimental extracts. Data are presented as median (minimum-maximum). Intergroup comparisons at each time point were performed using a nonparametric Mann–Whitney test. Differences were considered statistically significant at *p* < 0.05.

Animal Groups	Neutrophils, %			Macrophages, %			Lymphocytes, %			Fibroblasts, %		
	Day 1	Day 3	Day 7	Day 1	Day 3	Day 7	Day 1	Day 3	Day 7	Day 1	Day 3	Day 7
Control	95 (92–107)	80 (75–82)	58 (55–65)	2 (1–4)	15 (12–17)	18 (16–20)	3 (2–5)	5 (4–6)	3 (2–4)	0 (0–0)	5 (4–6)	15 (12–16)
“*Rotokan*”	88 (85–96) **	60 (55–65) **	30 (25–35) **	5 (4–6) **	22 (18–25) *	25 (22–28) **	4 (3–5)	7 (6–8) *	5 (4–6) *	0 (0–0)	3 (1–4) *	37 (26–43) **
S1	93 (87–96)	82 (77–83)	61 (57–66)	1 (0–6)	16 (13–18)	17 (17–19)	2 (2–4)	5 (3–7)	2 (1–5)	0 (0–0)	4 (2–6)	17 (14–18)
S2	91 (85–94)	82 (76–84)	59 (56–62)	3 (2–5)	14 (11–18)	16 (13–19)	2 (1–5)	6 (3–10)	4 (2–7)	0 (0–0)	4 (2–6)	13 (10–18)
S3	94 (90–101)	82 (73–90)	56 (50–64)	1 (0–5)	17 (11–19)	15 (11–18)	4 (1–8)	7 (3–12)	2 (0–5)	0 (0–0)	2 (0–6)	11 (7–15)
S4	98 (94–105)	77 (71–86)	58 (51–64)	3 (1–5)	16 (11–21)	17 (12–21)	2 (1–6)	4 (1–7)	1 (0–6)	0 (0–0)	4 (2–8)	18 (12–23)
S5	92 (91–103)	82 (75–88)	56 (54–63)	3 (1–7)	12 (9–18)	16 (12–21)	2 (0–6)	7 (2–10)	2 (1–6)	0 (0–0)	2 (1–7)	14 (9–16)
S6	95 (88–105)	81 (76–89)	57 (50–68)	1 (0–7)	14 (10–18)	20 (15–27)	2 (1–4)	4 (1–8)	2 (1–6)	0 (0–0)	4 (1–7)	12 (7–18)
S7	92 (85–101)	76 (71–82)	54 (49–61)	3 (1–5)	16 (7–23)	19 (15–27)	2 (1–6)	3 (2–7)	2 (1–5)	0 (0–0)	3 (2–7)	16 (11–21)
S8	91 (84–100)	84 (76–97)	61 (55–73)	1 (0–5)	14 (11–21)	21 (15–25)	2 (1–7)	6 (4–11)	5 (1–11)	0 (0–0)	7 (2–17)	19 (9–28)
S9	95 (87–107)	78 (71–92)	54 (43–61)	1 (0–5)	12 (8–17)	20 (12–25)	1 (0–6)	4 (2–8)	2 (0–9)	0 (0–0)	2 (0–8)	17 (10–26)
S10	93 (89–107)	84 (77–92)	67 (56–72)	1 (0–8)	14 (7–25)	19 (7–31)	2 (0–7)	3 (1–9)	1 (0–5)	0 (0–0)	6 (1–11)	17 (8–22)
S11	98 (87–110)	85 (77–91)	54 (49–65)	3 (0–6)	12 (8–17)	19 (13–25)	2 (0–6)	3 (1–7)	3 (0–6)	0 (0–0)	4 (1–7)	18 (11–23)
S12	100 (81–112)	84 (77–101)	52 (47–63)	3 (1–7)	12 (7–16)	14 (9–18)	2 (1–6)	3 (2–8)	2 (1–5)	0 (0–0)	2 (1–6)	17 (11–21)
S13	86 (82–94)	50 (45–54) *	28 (21–33) *	4 (3–7)	17 (13–20) *	23 (21–27)	3 (2–6)	4 (2–5) *	4 (2–7)	0 (0–0)	3 (1–4)	37 (26–43) *

* *p* < 0.05, ** *p* < 0.01 compared to the control group at the corresponding time point (in the case of “*Rotokan*”) and compared to “*Rotokan*” (in the case of compound S13).

**Table 3:** Dry extracts obtained from *S. officinale*.

Sample	Part of Plant	Solvent	Extract	Yield of the Extract, %
S1	Roots	Water	Crude	20.38 *
S2	Roots	40% ethanol	Crude	18.21
S3	Roots	70% ethanol	Crude	14.42
S4	Roots	Water	Polyphenolic fraction	8.62
S5	Roots	Water	Polysaccharide fraction	9.87
S6	Leaves	Water	Crude	23.12 *
S7	Leaves	40% ethanol	Crude	20.43
S8	Leaves	70% ethanol	Crude	16.93
S9	Leaves	Water	Polyphenolic fraction	13.54
S10	Leaves	Water	Polysaccharide fraction	7.51
S11	Flowers	Water	Crude	24.39
S12	Flowers	40% ethanol	Crude	21.38
S13	Flowers	70% ethanol	Crude	17.31

Note. *—due to the presence of mucilage, separation from the plant material was difficult.

## Data Availability

The data supporting the results of this study can be obtained from the corresponding authors upon reasonable request.

## Supplementary Materials

The following supporting information can be downloaded at: https://www.mdpi.com/article/10.3390/molecules31122159/s1, Figure S1: MRM chromatograms of quantified compounds in S9 sample. Neochlorogenic acid 1; Chlorogenic acid 2; Hyperoside 3; Isoquercitrin 4; Phlorizin 5; p-Coumaric acid 6; Ferulic acid 7; Caffeic acid 8; 3.4-Dihyrobenzoic acid 9; Rosmarinic acid 10; Kaempherol-3-O-glucoside 11; Figure S2: MRM chromatograms of analytical standards of quantified polyphenolic compounds. Neochlorogenic acid 1; Chlorogenic acid 2; Hyperoside 3; Isoquercitrin 4; Phlorizin 5; p-Coumaric acid 6; Ferulic acid 7; Caffeic acid 8; 3.4-Dihyrobenzoic acid 9; Rosmarinic acid 10; Kaempherol-3-O-glucoside 11; Figure S3: MRM chromatograms of quantified amino acids in S12 sample. Alanine 1; Serine 2; Proline 3; Valine 4; Leucine 5; Isoleucine 6; Aspartic acid 7; Lysine 8; Glutamic acid 9; Phenylalanine 10; Arginine 11; Threonine 12; Methionine 13; Histidine 14; Figure S4: MRM chromatograms of analytical standards of quantified amino acids. Alanine 1; Serine 2; Proline 3; Valine 4; Leucine 5; Isoleucine 6; Aspartic acid 7; Lysine 8; Glutamic acid 9; Phenylalanine 10; Arginine 11; Threonine 12; Methionine 13; Histidine 14; Figure S5: Positive ion mode scan chromatograms of PAs from sample S1. Total ion scan chromatogram a), extracted ion chromatograms at specific *m*/*z* values of 300.2 (b), 316.2 (c), 342.2 (d) and 358.2 (e); Figure S6: MRM mode chromatograms of sample S1. Summed up ion transitions of 300 > 94 and 300 > 138 at a); 316 > 94 and 316 > 172 at b); 342 > 94, 342 > 118, 342 > 120, 342 > 137 and 342 > 180 at c); 358 > 137, 358 > 180 and 358 > 214 at d); Figure S7: MRM chromatograms of PAs standards. Intermedine a); Lycopsamine b); Intermedine-N-oxide c); Lycopsamine-N-oxide d).
