# Supplementary material for: Organ-Specific Phytochemical Profiles, Wound-Healing and Hemostatic Activities of Symphytum officinale Aerial Parts and Roots with Differential Pyrrolizidine Alkaloid Content
Source: Molecules. 2026 Jun 18;31(12):2159. doi: 10.3390/molecules31122159 (PMC13306179; doi:10.3390/molecules31122159)
Supplement: Supplementary file 1 [file molecules-31-02159-s001.zip › molecules-4368129-supplementary.pdf]

Supplementary Materials

Organ-Specific Phytochemical Profiles, Wound-Healing and Hemostatic Activities of *Symphytum officinale* Aerial Parts and Roots with Differential Pyrrolizidine Alkaloid Content

Getter Dolgošev [1,*], Yurii M. Kolesnyk [2], Olha Hancheva [2], Oleksandr Panasenko [2], Andrii Kaplaushenko [2], Roman Shcherbyna [2], Valdas Jakštas [3,4], Vaidotas Žvikas [3], Ivo Laidmäe [1], Jyrki Heinämäki [1], Oleh Koshovyi [1,2,*] and Ain Raal [1]

[1] Institute of Pharmacy, Faculty of Medicine, University of Tartu, 50411 Tartu, Estonia; ivo.laidmae@ut.ee (I.L.); jyrki.heinamaki@ut.ee (J.H.); ain.raal@ut.ee (A.R.)

[2] Zaporizhzhia State Medical and Pharmaceutical University, 69035 Zaporizhzhia, Ukraine; kympat83@gmail.com (Y.M.K.); ganchevaolga1@gmail.com (O.H.); panasenko.o.i@zsmu.edu.ua (O.P.); kaplaushenko@ukr.net (A.K.); rscherbyna@gmail.com (R.S.)

[3] Institute of Pharmaceutical Technologies, Lithuanian University of Health Sciences, LT-44307 Kaunas, Lithuania; valdas.jakstas@lsmu.lt (V.J.); vaidotas.zvikas@lsmu.lt (V.Ž.)

[4] Department of Pharmacognosy, Lithuanian University of Health Sciences, LT-44307 Kaunas, Lithuania

* Correspondence: getter.dolgosev@ut.ee (G.D.); oleh.koshovyi@ut.ee (O.K.); Tel.: +372-502-7574 (G.D. & O.K.)

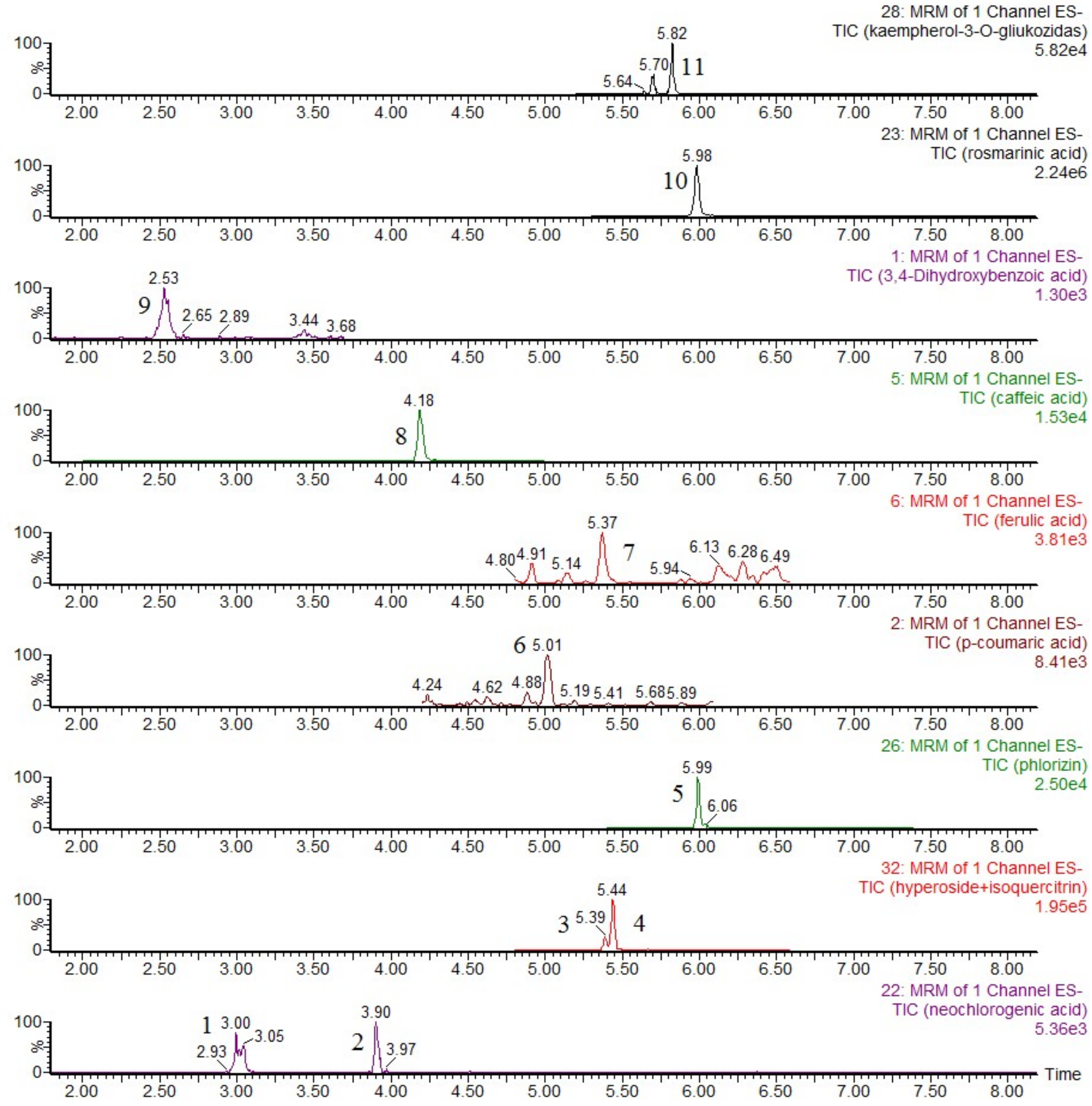

Figure S1. MRM chromatograms of quantified compounds in S9 sample. Neochlorogenic acid 1; Chlorogenic acid 2; Hyperoside 3; Isoquercitrin 4; Phlorizin 5; p-Coumaric acid 6; Ferulic acid 7; Caffeic acid 8; 3.4-Dihyrobenzoic acid 9; Rosmarinic acid 10; Kaempherol-3-O-glucoside 11.

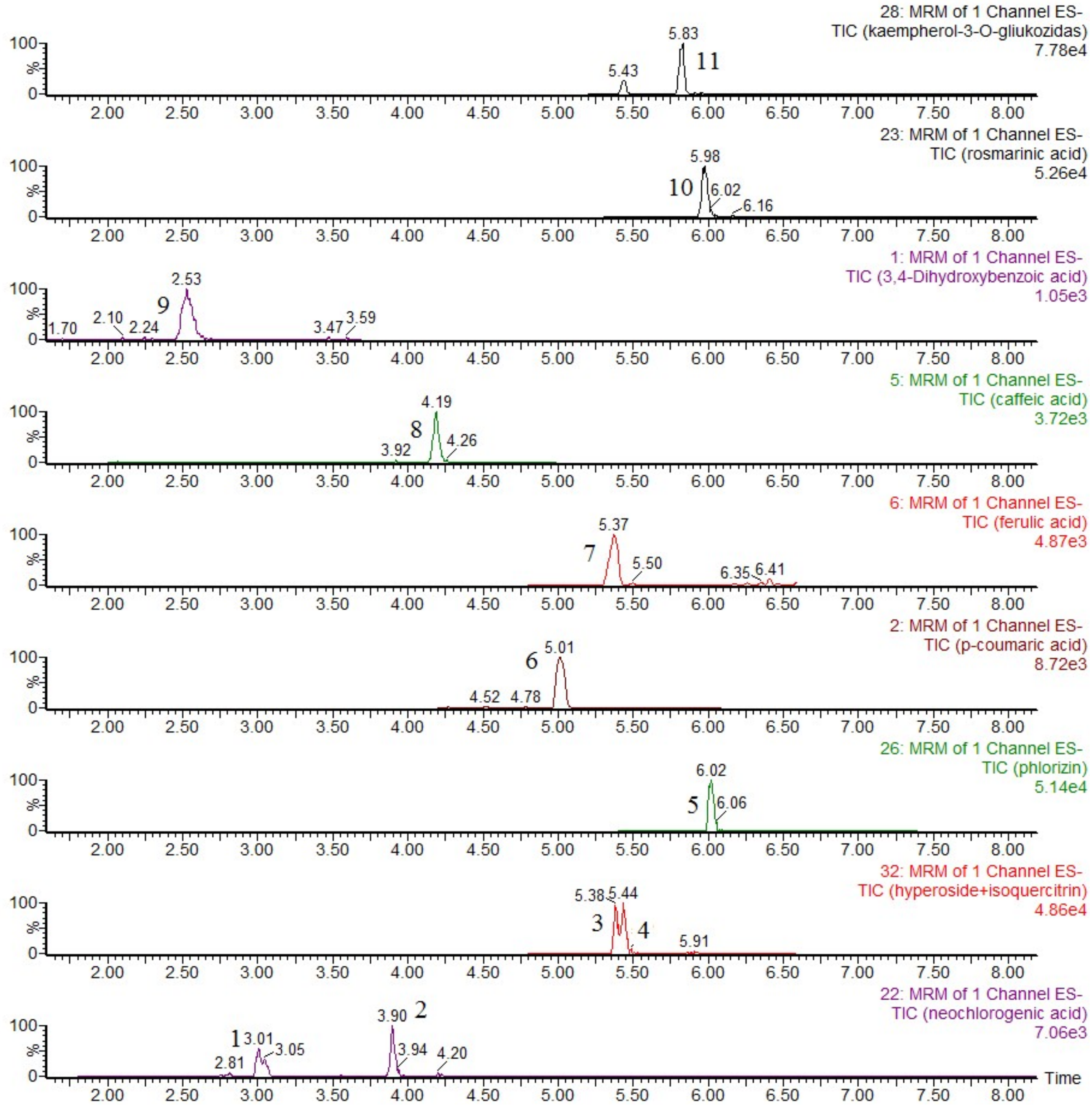

Figure S2. MRM chromatograms of analytical standards of quantified polyphenolic compounds. Neochlorogenic acid 1; Chlorogenic acid 2; Hyperoside 3; Isoquercitrin 4; Phlorizin 5; p-Coumaric acid 6; Ferulic acid 7; Caffeic acid 8; 3.4-Dihyrobenzoic acid 9; Rosmarinic acid 10; Kaempherol-3-O-glucoside 11.

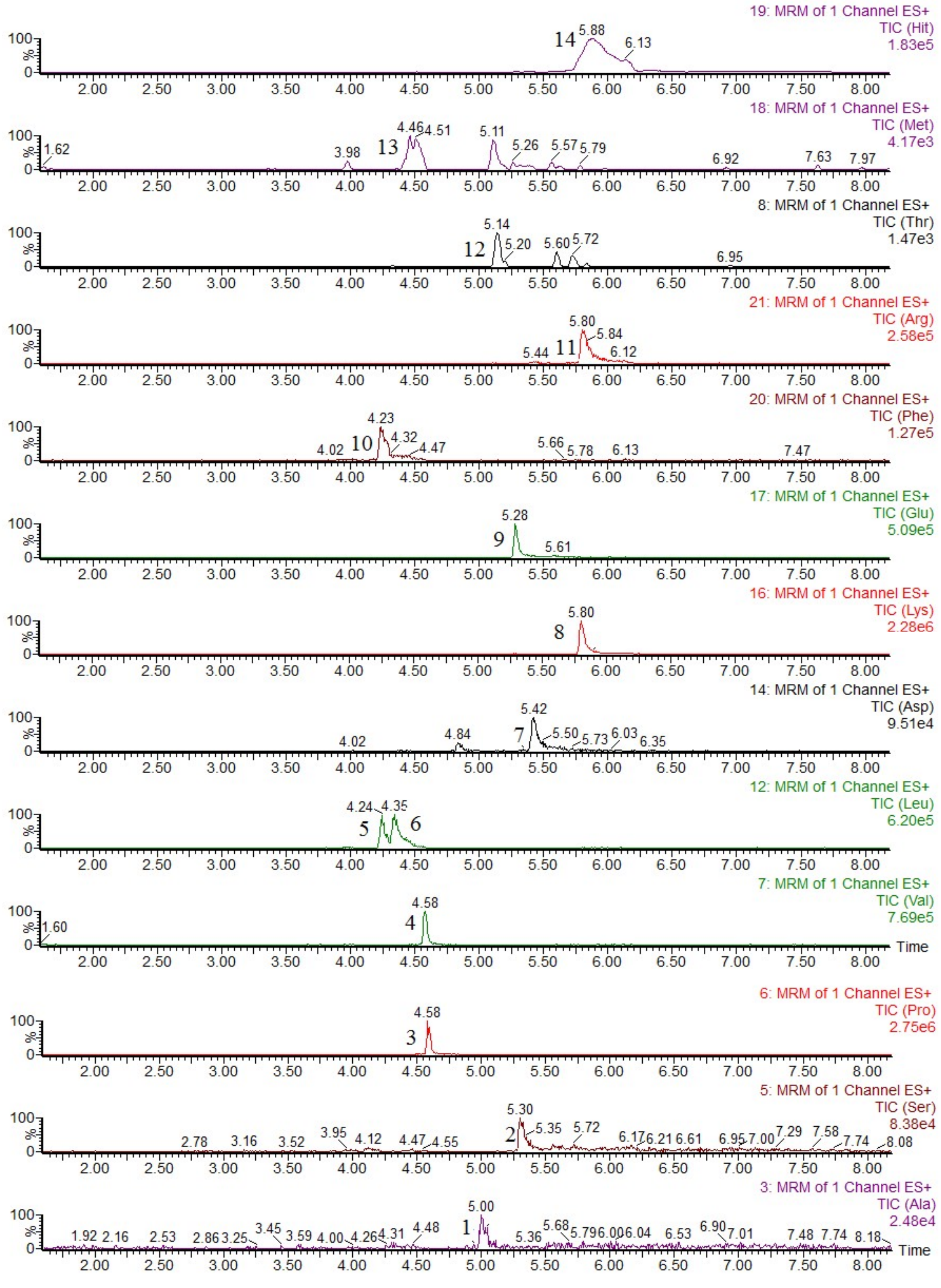

Figure S3. MRM chromatograms of quantified amino acids in S12 sample. Alanine 1; Serine 2; Proline 3; Valine 4; Leucine 5; Isoleucine 6; Aspartic acid 7; Lysine 8; Glutamic acid 9; Phenylalanine 10; Arginine 11; Threonine 12; Methionine 13; Histidine 14.

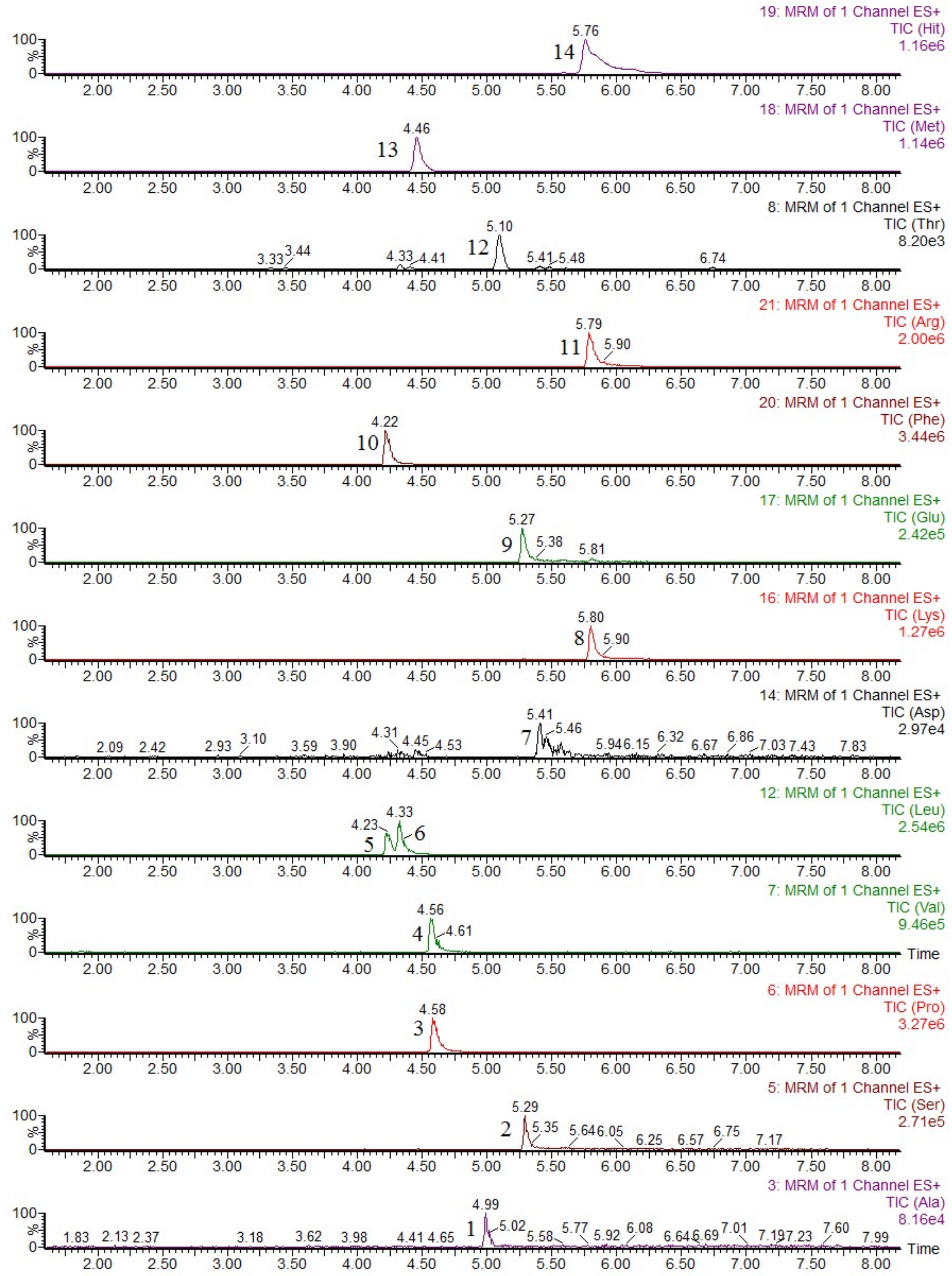

Figure S4. MRM chromatograms of analytical standards of quantified amino acids. Alanine 1; Serine 2; Proline 3; Valine 4; Leucine 5; Isoleucine 6; Aspartic acid 7; Lysine 8; Glutamic acid 9; Phenylalanine 10; Arginine 11; Threonine 12; Methionine 13; Histidine 14.

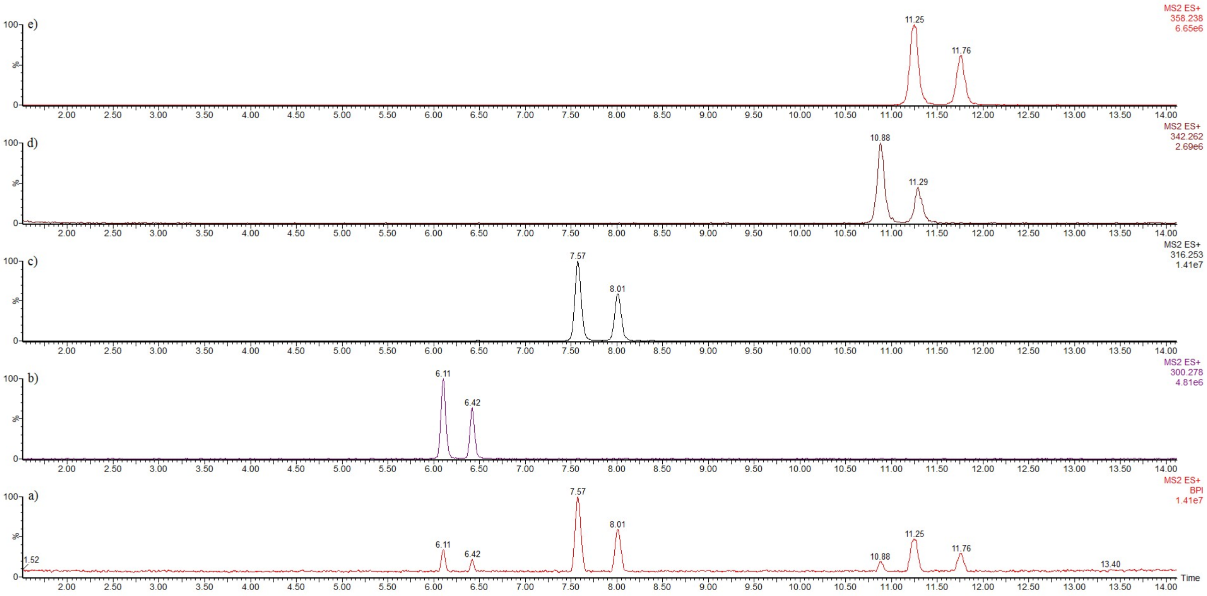

Figure S5. Positive ion mode scan chromatograms of PAs from sample S1. Total ion scan chromatogram a), extracted ion chromatograms at specific m/z values of 300.2 (b), 316.2 (c), 342.2 (d) and 358.2 (e).

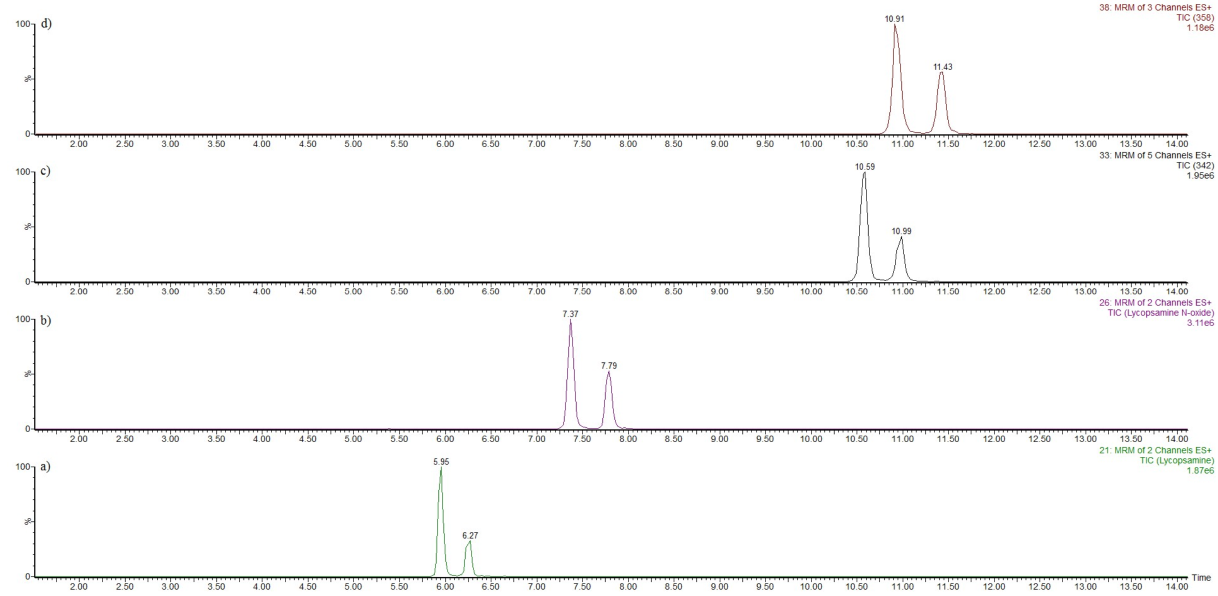

Figure S6. MRM mode chromatograms of sample S1. Summed up ion transitions of 300 > 94 and 300 > 138 at a); 316 > 94 and 316 > 172 at b); 342 > 94, 342 > 118, 342 > 120, 342 > 137 and 342 > 180 at c); 358 > 137, 358 > 180 and 358 > 214 at d).

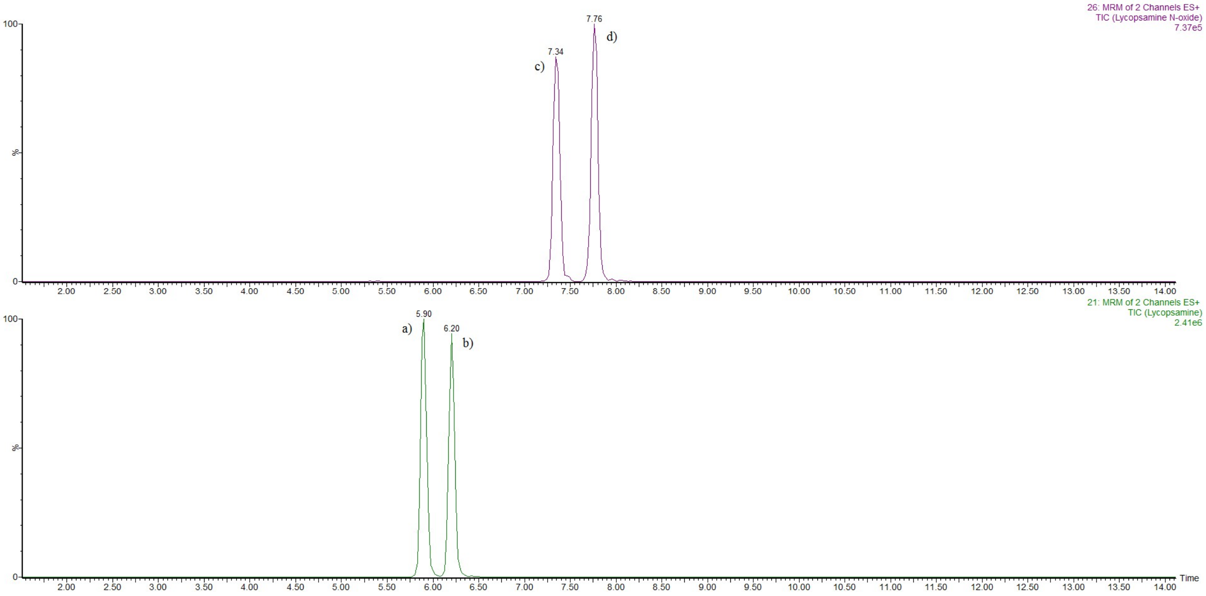

Figure S7. MRM chromatograms of PAs standards. Intermedine a); Lycopsamine b); Intermedine-N-oxide c); Lycopsamine-N-oxide d).
